# Induction Heating in Nanoparticle Impregnated Zeolite

**DOI:** 10.3390/ma13184013

**Published:** 2020-09-10

**Authors:** Irene Morales, Marta Muñoz, Catia S. Costa, Jose Maria Alonso, João Miguel Silva, Marta Multigner, Mario Quijorna, M. Rosário Ribeiro, Patricia de la Presa

**Affiliations:** 1 Instituto de Magnetismo Aplicado, UCM-ADFI-CSIC, A6 22,500 Km, 28230 Las Rozas, Spain; irenemorales@ucm.es (I.M.); jm.a.r.0@csic.es (J.M.A.); maquijor@ucm.es (M.Q.); 2 Departamento de Matemática Aplicada, Ciencia e Ingeniería de los Materiales y Tecnología Electrónica, Universidad Rey Juan Carlos, 28933 Madrid, Spain; marta.munoz@urjc.es (M.M.); marta.multigner@urjc.es (M.M.); 3 Centro de Química Estrutural, Instituto Superior Técnico, Universidade de Lisboa, 1049-001 Lisboa, Portugal; catia.s.costa@tecnico.ulisboa.pt (C.S.C.); jmsilva@deq.isel.ipl.pt (J.M.S.); rosario@tecnico.ulisboa.pt (M.R.R.); 4 Instituto de Ciencias de Materiales de Madrid, ICMM-CSIC, 28049 Madrid, Spain; 5 Instituto Superior de Engenharia de Lisboa, Instituto Politécnico de Lisboa, 1959-007 Lisboa, Portugal; 6 Departamento Física de Materiales, Universidad Complutense de Madrid, 28040 Madrid, Spain

**Keywords:** magnetic nanoparticles, catalytic cracking, induction heating, zeolite

## Abstract

The ultra-stable Y (H-USY) zeolite is used as catalyst for the conversion of plastic feedstocks into high added value products through catalytic cracking technologies. However, the energy requirements associated with these processes are still high. On the other hand, induction heating by magnetic nanoparticles has been exploited for different applications such as cancer treatment by magnetic hyperthermia, improving of water electrolysis and many other heterogeneous catalytic processes. In this work, the heating efficiency of γ-Fe_2_O_3_ nanoparticle impregnated zeolites is investigated in order to determine the potential application of this system in catalytic reactions promoted by acid catalyst centers under inductive heating. The γ-Fe_2_O_3_ nanoparticle impregnated zeolite has been investigated by X-ray diffraction, electron microscopy, ammonia temperature program desorption (NH_3_-TPD), H_2_ absorption, thermogravimetry and dc and ac-magnetometry. It is observed that the diffusion of the magnetic nanoparticles in the pores of the zeolite is possible due to a combined micro and mesoporous structure and, even when fixed in a solid matrix, they are capable of releasing heat as efficiently as in a colloidal suspension. This opens up the possibility of exploring the application at higher temperatures.

## 1. Introduction

The increase in plastic production (10% every year since 1950) with an increasingly shorter use causes serious environmental problems [1,2]. Conventional recycling pathways, such as mechanical recycling or incineration, exhibit some limitations. The first route cannot be applied to all types of plastic waste, requiring single plastics feedstocks and efficient sorting techniques, while the second one releases toxic compounds to the atmosphere, leading to environmental pollution. Therefore, chemical recycling is getting increasing attention as an effective process to convert plastic waste into chemicals or fuels, thus replacing fossil resources for chemical production [3].

A very effective route for the conversion of waste plastic materials into high added value products involves the catalytic cracking of polymer chains promoted by acid catalysts [4]. Several publications refer to zeolite materials as effective catalysts for this type of application [5,6,7], since they have high thermal stability, strong acidity, a unique porous structure, and a highly crystalline framework [8]. However, their microporous nature also presents some limitations, especially in what concerns mass transfer limitations of bulky molecules inside the pores [9]. These constraints can be reduced through the use of mesoporous structures with acidic character or hierarchical zeolites exhibiting both micro and mesoporosity [10,11]. Among the various types of zeolite structures (ex: H-ZSM-5, H-MOR, H-FER), H-USY zeolite stands out for its greater accessibility. Its large porous structure (formed by an interconnected supercage with 1.3 nm of internal diameter, which is accessed by smaller channel openings of 0.7 nm) and high external surface area favor cracking reactions either on the surface or inside of the pores. Along with the accessibility, the acidy of the H-USY zeolite, which can be controlled by the Si/Al ratio, also plays an essential role in the reaction. These characteristics made H-USY a very promising catalyst for the conversion of waste plastic materials. Its potential for this type of application was already proved by several authors in literature [12,13,14,15,16]. Despite the promising results described in the literature for plastic waste conversion through cracking technologies with acidic catalyst under either inert or reducing atmosphere, energy requirements are still an issue.

On the other hand, the use of nanoparticles for induction heating under radiofrequency fields has been shown to be very useful for different catalytic processes [17] such as heterogeneous catalysis, chemical reactions in organic or inorganic media [18,19], water electrolysis [20] and others [21,22,23,24]. The induction heating by magnetic nanoparticles is mainly focused on the advantages of this approach in terms of process intensification, energy efficiency, etc. It is a technology that could reduce the problems of energy transfer inefficiency and heat dissipation phenomena. It offers unique solutions compared to the catalytic transformations by conventional heating methodologies because it can overcome heat transfer limitations, such as slow heating/cooling speeds, non-uniform heating environments, low energy efficiency, etc.

Superparamagnetic nanoparticles, specifically maghemite (γ-Fe_2_O_3_), have been proved to have high specific loss power (SLP) generating thermal energy under alternating magnetic fields [25,26,27,28,29]. The heating mechanism of these nanoparticles depends on two relaxation mechanisms: Néel relaxation (the magnetization rotates inside the monodomain) and Brown relaxation (the particle rotates physically) [30]. If the support for the catalytic process is a solid matrix, like in the case of zeolite for cracking applications, the main mechanism process is Néel since the Brownian relaxation is canceled. If the only process allowed is Néel relaxation, then it must exist as a compromise between size and magnetic anisotropy of the materials: if the particles are small with low blocking temperature, the saturation is low and the frequency required is normally high, making the heating efficiency poor and energetically expensive; if the particles are large, the blocking temperature and saturation magnetization are higher but the largest particles that relax by the Brownian process would not contribute to the heating. Therefore, it is a challenge to find out which magnetic nanomaterial would be the proper one to induce efficient heating in a solid matrix. Metallic iron nanoparticles of a small size would be the best candidate because the saturation magnetization is high and the magnetic anisotropy is low, making them the ideal material for this application. However, Fe nanoparticles have the drawback that they oxide easily to other non-magnetic materials such as hematite or wüstite [31,32] giving place to a core-shell structure and impelling an efficient conversion of the electromagnetic energy into heat. One way to obtain metallic Fe nanoparticles is by means of the design of iron oxide impregnated zeolite followed by in situ reduction in nitrogen atmosphere. The hydrocracking method is ideal because the iron oxide nanoparticles impregnated in the zeolite can be reduced to metallic iron nanoparticles with the advantages that once the nanoparticles are dispersed in the zeolite matrix they cannot aggregate and, besides, the oxidation to non-magnetic oxide is prevented.

Therefore, the aim of this work is to evaluate the heating efficiency of γ-Fe_2_O_3_ nanoparticles when impregnated in a zeolite solid matrix and to test the reduction temperature under hydrogen for the magnetic nanoparticles and the nanoparticle impregnated zeolites. It matters that the γ-Fe_2_O_3_ nanoparticles can produce heat in the solid matrix because it will be the first step for the conversion of γ-Fe_2_O_3_ into metallic Fe nanoparticles, which can further enhance the heating efficiency. These first results show that zeolite H-USY (40) with a Si/Al of 40 can internalize γ-Fe_2_O_3_ nanoparticles of 11 nm, and these nanoparticles are able to induce heat under radiofrequency field even when Brownian relaxation is not present.

## 2. Materials and Methods

### 2.1. Synthesis of Magnetic Nanoparticles

A modified co-precipitation synthesis method [27] was chosen for the synthesis of γ-Fe_2_O_3_ nanoparticles because this method allows one to produce large volumes at low cost and, in addition, it is possible to control the particle size distribution [33]. This method consists in the synthesis of Fe_3_O_4_ nanoparticles of 10–12 nm and the subsequent reduction to γ-Fe_2_O_3_. Briefly, solutions of 24.3 g of FeCl_3_·6H_2_O and 10.8 g of FeCl_2_·4H_2_O in 43 and 45 mL of distilled water, respectively, were prepared. Later, they were dissolved in 400 mL of distilled water under magnetic stirring. A solution of 75 mL at 25% NH_4_OH was prepared and slowly added to the mixture. The precipitation of Fe_3_O_4_ nanoparticles of 10–12 nm was obtained by heating the solution up to 90 °C for 60 min under N_2_ flux [34,35]. The nanoparticles were washed three times with distilled water by magnetic decantation.

Once the Fe_3_O_4_ (Fe II, FeIII) nanoparticles were obtained they were oxidized to γ-Fe_2_O_3_ (Fe III). Briefly, 300 mL of HNO_3_ (2 M) was added to the nanoparticles and stirred for 15 min. Then, the supernatant was removed by magnetic decantation and 75 mL of Fe(NO_3_)_3_·9H_2_O at 1 M were added and heated until boiling for 30 min. After cooling down the mixture and removing the supernatant, 300 mL of HNO_3_ at 2 M was added drop by drop while stirring the mixture for 15 min. This last procedure allows for the dissolving of the smallest nanoparticles and recrystallizing the larger ones. Finally, the γ-Fe_2_O_3_ nanoparticles were separated by magnetic decantation and washed with acetone three times. The acetone was evaporated in a rotary evaporator. Finally, the sample was dispersed in distilled water.

### 2.2. Zeolite and Magnetic Nanoparticles

Zeolite: Two commercial zeolites in powder form supplied by Zeolyst were used as supports for the magnetic nanoparticle impregnation: H-USY (15) and H-USY (40) with a Si/Al of 15 and 40, respectively.

Zeolite impregnated with magnetic nanoparticles: To optimize the catalytic properties keeping high heating powers, supported magnetic nanoparticles-catalysts were prepared by incipient wetness impregnation method from the two H-USY zeolites. In this case, the magnetic nanoparticle-based zeolites were prepared according to the incipient wetness impregnation method as follows: an aqueous magnetic colloid of γ-Fe_2_O_3_, with a volume closer to that zeolite pore saturation of 1.7 mL and previously dispersed by ultrasound, was added drop by drop to 1 g of zeolite. Saturation concentration of the H-USY zeolite was reached by mechanical dispersion with a glass rod. After the impregnation procedure, the wet powder is left to rest for a few hours and the samples were afterward air-dried at 80 °C during 24 h.

### 2.3. Structural and Compositional Characteriztion

The study of the crystalline structure of the zeolite was done by X-ray diffraction (XRD, PANalytical X’Pert MPD, Cambridge, UK) using a PANalytical X’Pert MPD diffractometer with a copper anode (λ = 1.5418 Å) and a graphite monochromator. The analysis of diffractograms was carried out using the X’Pert HighScore Plus program.

The textural properties of parent H-USY zeolites and the corresponding nanoparticle impregnated zeolites were evaluated by N_2_ adsorption measurements. The experiments were performed at −196 °C using an Autosorb IQ apparatus from Quantachrome (Boynton Beach, FL, USA). Before the adsorption, the materials were degassed under vacuum at 90 °C for 1 h and then heated at 350 °C for 5 h. External surface area (Sext) and micropore volume (Vmicro) were calculated using the t-plot method, whereas the total pore volume was determined from the adsorbed volume of nitrogen at a relative pressure (P/P0) of 0.95. The difference between V_total_ and V_micro_ gives the mesopore volume (V_meso_).

The acidic properties of the parent and nanoparticle impregnated zeolites were carried out by ammonia temperature program desorption (NH_3_-TPD). The samples were pre-treated under a helium atmosphere at 350 °C for 1 h and then cooled down to 125 °C and ammonia-saturated in a stream of 15% NH_3_/He at a flow of 30 mL/min, for 1 h. Prior to the desorption step, the samples were outgassed under helium (He) at 125 °C during 30 min, to remove the physiosorbed-ammonia. Chemisorbed-NH_3_ was desorbed at a heating rate of 10 °C/min until 700 °C and the amount of ammonia desorbed in the effluent stream was detected by a thermal conductivity detector (TCD, VICI Valco Instruments, Hoston, TX, USA). The total acidity of the catalyst was obtained by integrating the area under the desorption curve.

The final concentration of γ-Fe_2_O_3_ nanoparticles in solution was measured by a Perkin Elmer IPC (Waltham, MA, USA) plasma emission spectrometer model Optima 2100 DV, obtaining the Fe concentration. It was performed by adding a few drops of HCl to 25 µL of the solution with nanoparticles and flushing up to 25 mL with distilled water. The final Fe concentration was 60.5 mg/mL which implies that the nanoparticle impregnated zeolite was around 14.7 wt.% of γ-Fe_2_O_3_.

For structural characterization of the nanoparticles, a JEOL JEM1010 electronic transmission microscope (TEM) operating at 100 kV, from the Autonomous University of Madrid (Madrid, Spain) was used. The average size and its distribution were obtained using the Fiji-win32 software (Madison, WI, USA) counting more than 800 particles.

The zeolites H-USY (15) and H-USY (40) and the nanoparticle impregnated zeolites have been characterized using images obtained by TEM, with the JEOL JEM 2100F microscope with an intensity of 200 keV, from the National Center for Electron Microscopy, Complutense University of Madrid (Madrid, Spain).

A thermogravimetric analysis (TGA) of nanoparticles, zeolite and nanoparticle impregnated zeolite was performed on a Cahn D-200 thermobalance, Complutense University of Madrid, Madrid, Spain. The study of the samples has been carried out in a reducing atmosphere of 0.3 atm He/0.2 atm H_2_ and linear temperature progression regime, at 6 °C/min, from room temperature up to 900 °C. The nanoparticle impregnated zeolite was previously degassed by heating up to 900 °C in 0.5 atm He and then cooled down to room temperature. According to the literature, the ultra-stabilized Y zeolite can maintain the micro porosity until a temperature near 900 °C [36].

### 2.4. Magnetic Characterization

The magnetic characterizations of the nanoparticles were performed in a SQUID Quantum Design (San Diego, CA, USA). Zero field cooled (ZFC) and field cooled (FC) curves were measured from 5 to 300 K at 100 Oe. Hysteresis loops have been measured at 5 and 300 K with 5 T maximum applied field.

In addition, the calorimetric properties were characterized in a Magnetherm 1.5 device (Nanotherics, Warrington, UK). The system allows for working with ten different resonance frequencies to study the effect of the field amplitude and the frequency on the heating release of the nanoparticles. The coil temperature was maintained at 16 °C with a LAUDA Alpha RA12 water bath refrigerator peristaltic device. The temperature increase of the magnetic colloid has been measured by an optical probe and recorded in a computer as a function of time for each field. In a similar way, the temperature increase of the nanoparticle impregnated zeolite was measured with a thermographic camera FLIR E53, field of vision 24° × 18° Lens, and 240 × 180 pixels resolution and registered in the computer.

The heating efficiency is evaluated by means of the SLP as:

(1) $$\mathrm{SLP}=\frac{C_{liq}}{\left[Fe\right]}\frac{\Delta T}{\Delta t}$$

where, C_liq_ is the heat capacity of water (4.185 J/gK), [Fe] is the percentage weight of iron (0.06 wt.%) and ∆T/∆t is the initial slope of the heating curve. Before measuring the slope, temperature was registered 30 s with field off in order to have a base line for the slope calculation. The slope of the heating curve is calculated in the first 30–50 s after the field was turned on [37,38].

## 3. Results and Discussion

**Figure 1:**
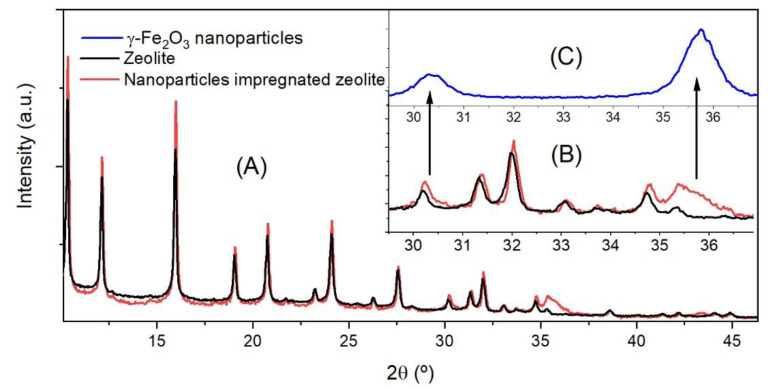
(**A**) XRD pattern of H-USY (40) zeolite (black lines) and nanoparticles impregnated H-USY (40) zeolite (red lines). (**B**) The zeolite and nanoparticles impregnated zeolite in the range from 29.5° to 36.9°. (**C**) The inset shows a detail of the XRD diffraction pattern of γ-Fe_2_O_3_ nanoparticles in the same range; the arrows point out to the 36% and 100% intensity peaks of γ-Fe_2_O_3_.

Figure 1 shows the X-ray diffraction pattern of H-USY (40) zeolite, and γ-Fe_2_O_3_ impregnated H-USY (40) together with a detail of the diffraction pattern of γ-Fe_2_O_3_ nanoparticles. The complete diffraction pattern of maghemite is shown in Figure S1 (see Supplementary Material); the crystallite size calculated by the Scherrer formula is 11.1 nm, the peaks are wide and of little intensity because of the small size of the nanoparticles. In Figure 1A, the zeolite peaks at 2θ = 6.2, 10.3, 12.1, 15.9, 18.9, 20.7, 24.0 and 27.5° are typical of a supercrystalline Y type zeolite [39]. Regarding the γ-Fe_2_O_3_ impregnated zeolite, the amount of nanoparticles is only 15 wt.% which makes it difficult to observe the corresponding diffraction peaks because these are wide and have much lower intensity compared to the intense peaks of the highly crystalline zeolite. However, the 100% peak of the maghemite is observed in the nanoparticle impregnated zeolite. The H-USY (40) zeolite has a diffraction peak of 0.6% intensity at 2θ = 35.4°, which is close to the 100% peak of γ-Fe_2_O_3_ at 2θ = 35.7°. Figure 1B shows the overlap of both peaks and it is possible to observe the 100% peak of the γ-Fe_2_O_3_ as a shoulder of the zeolite diffraction peak. The Figure 1C shows a detail of the diffraction pattern of γ-Fe_2_O_3_ nanoparticle in the same angle range of the nanoparticle impregnated zeolite, it is worth noting that the wide of the 100% peak of the γ-Fe_2_O_3_ fits pretty well with the shoulder wide of the nanoparticle impregnated zeolite. Therefore, XRD confirms the presence of γ-Fe_2_O_3_ in the zeolite.

**Figure 2:**
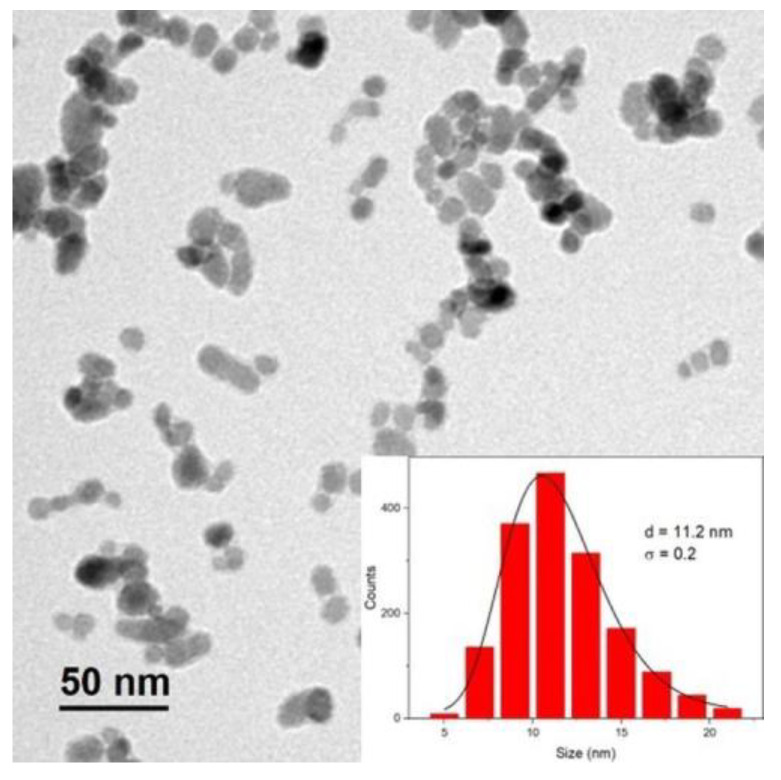
TEM image and the corresponding histogram of the γ-Fe_2_O_3_ nanoparticles (inset).

Figure 2 shows TEM images of the nanoparticles together with the size distribution fitted to a lognormal function. The histogram gives a mean particles of d = 11.2 nm and polydispersity degree (standard deviation/mean size) σ = 0.2, as expected [27], which also fits with the crystallite size determined by XRD. In this type of synthesis by co-precipitation without surfactants, the nanoparticle size is controlled by different parameters like the nature of the basis used in the synthesis, the addition rate and subsequent acid treatment [40], where the smallest particles are dissolved contributing to the recrystallization of the largest one, thereby improving the magnetic properties.

**Figure 3:**
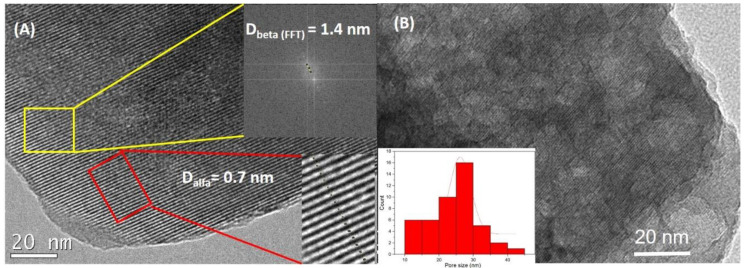
TEM images of (**A**) H-USY (15) with interplanar distance 1.4 nm determined by FFT and porous size of 0.7 nm and (**B**) H-USY (40) with mesoporous of 26 ± 6 nm mean size.

The TEM micrographs of H-USY (15) and H-USY (40) are shown in Figure 3. As can be seen in Figure 3A, the H-USY (15) is a well-ordered structure with the basic sodalite structural units assembled to form spherical supercages showing a diameter of 1.4 nm and an aperture of 0.7 nm [39]. On the other hand, H-USY (40) shows the same sodalite structure (it can be seen with more detail in Figure S2 of the Supplementary Material) besides lighter zones with a mean size of 26 ± 6 nm (see Figure 3B), these lighter zone can be associated to the mesopores of the zeolites. According to the literature [41], the appearance of mesoporosity in this type of zeolites with high Si/Al ratios is a result of the preparation method used. The H-USY (40) is obtained by successive dealumination treatments of mother zeolite H-USY (2.5). During this process Al species are removed from the zeolite structure leading to the formation of mesopores.

**Figure 4:**
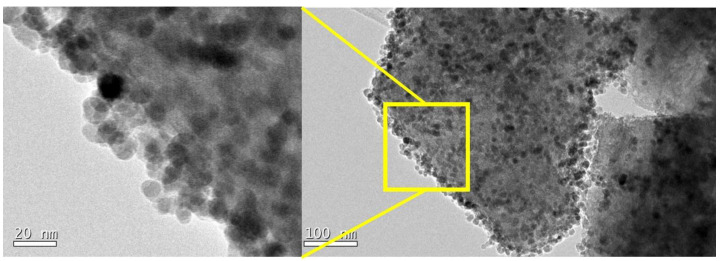
Nanoparticles impregnated zeolites H-USY (15). All the nanoparticles are on the surface. The micrograph on the left is the amplification of the yellow marked zone.

The TEM images of the γ-Fe_2_O_3_ impregnated H-USY (15) zeolite are shown in Figure 4. It is observed that all the NPs are located on the external surface of the zeolite probably because of the small pore size of H-USY (15). As mentioned previously, γ-Fe_2_O_3_ particles present an average size of 11.2 nm which is difficult for the internalization of the nanoparticles in the supercages of 1.4 nm in size.

**Figure 5:**
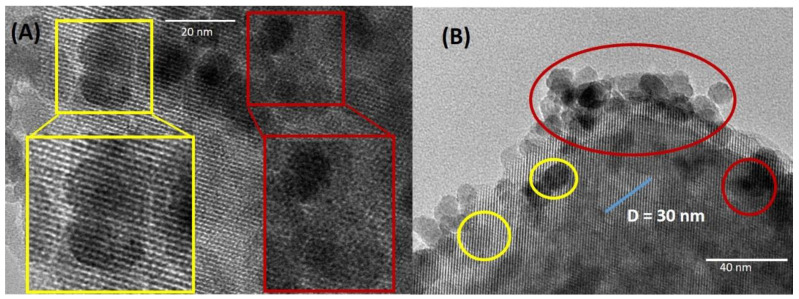
Nanoparticles impregnated zeolite H-USY (40). (**A**) TEM image of nanoparticle impregnated zeolite, the yellow frame indicates de nanoparticles crossed by the light lines (separated 1.6 nm) and the red frame shows only Fe_2_O_3_ nanoparticles. (**B**) A mesopores of 30 nm and several particles below the surface (in yellow) and above the surface (in red).

On the other hand, the γ-Fe_2_O_3_ impregnated H-USY (40) zeolite shows a completely different behavior. The TEM images in Figure 5A show γ-Fe_2_O_3_ nanoparticles (dark spheres) crossed by lighter lines separated by 1.6 nm, the characteristic features of H-USY (see Figure S3). The zeolite is a silicatoaluminates basically composed by Si and Al, chemical elements with atomic numbers Z 14 and 13, respectively, much lighter than Fe with Z = 26. In the transmitted-light microscope, variation of intensity within an image is caused by differences in the absorption of photons within different regions of the specimen and it depends on the atomic number Z: the higher the Z the lower the transmitted light. In the zeolite, the light can be transmitted through the micropores (white lines in Figure 5A), whereas the atomic arrangement of Si and Al appears as dark lines. On the contrary, γ-Fe_2_O_3_ has a compact packing cubic cell. The incident light on γ-Fe_2_O_3_ will not be transmitted due to the high Z of Fe and the high density of the packing, whereas the incident light on zeolite will be transmitted further due to its sodalite structure. Therefore, in the γ-Fe_2_O_3_ impregnated H-USY (40) there are nanoparticles below the surface (yellow frame Figure 5A) and above the surface (red frame Figure 5A).

In Figure 5B, a mesoporous of 30 nm in size is pointed out in addition to several particles crossed by the light lines (for more examples see Figure S4). The fact that the nanoparticles appear crossed by the supercages of the zeolite means that the nanoparticles are below the supercages. These images show that not all the nanoparticles are on the surface, as in the case of H-USY (15), some of them are below the surface and could be inside the zeolite. Accordingly, the TEM images for the γ-Fe_2_O_3_ impregnated H-USY (40) zeolite, the magnetic nanoparticles are located at the surface and could be also inside the mesopores.

The textural properties of parent and nanoparticle impregnated zeolites are displayed in Table 1. Data reveal that H-USY (40) presents the highest external surface area (S_ext_) and mesoporous volume (V_meso_), as a result of the post-synthesis dealumination treatments which led to the formation of a secondary mesoporosity. After nanoparticle impregnation, a decrease of the external area and the porous volume is observed for both H-USY zeolites. Other authors have also reported a reduction of the surface area and blockage of zeolite micropores by Ni particles [42,43]. The observed effect was larger when the metal particles were deposited on the external surface of the zeolites. In this work H-USY (15) shows the largest effect which may suggest that the magnetic nanoparticles are, in this case, located on the external surface thus, corroborating TEM data.

The acidic properties of parent and nanoparticle impregnated H-USY (15) and H-USY (40) zeolites are also exhibited in Table 1. The results show that an increase of the Si/Al ratio leads to a decrease of the total acidity, which is due to the decrease of aluminum species in the zeolite structure [44]. On the other hand, the subsequent addition of magnetic nanoparticles to the parent zeolites does not affect considerably the acidity of the catalysts, neither in terms of number, nor of the strength of the centers.

**Figure 6:**
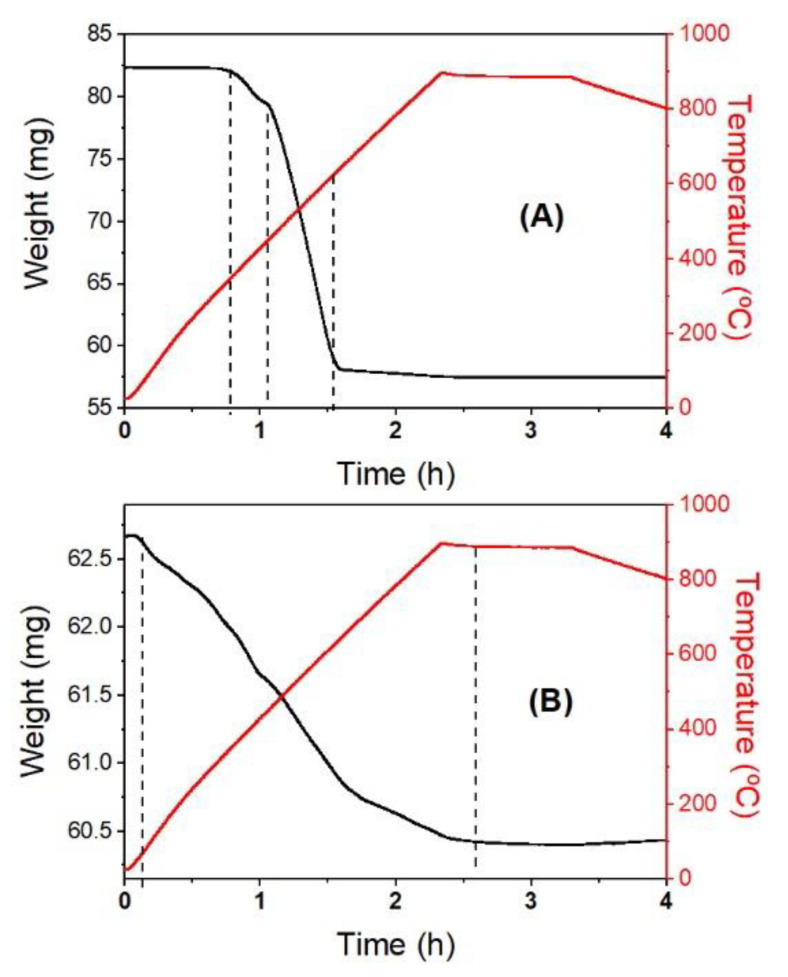
(**A**) Thermogravimetric analysis (TGA) of nanoparticles γ-Fe_2_O_3_. (**B**) TGA of nanoparticles impregnated zeolite H-USY (40). Black lines: weight vs. time. Red line: temperature vs. time. The dotted lines indicate the regions where reduction processes take place.

Since catalytic cracking reactions may be also carried out in a reducing atmosphere, it is important to investigate how magnetic oxides behave under such conditions. The results obtained by TG analysis of γ-Fe_2_O_3_ and of γ-Fe_2_O_3_ impregnated H-USY (40) zeolite under H_2_ atmosphere are shown in Figure 6. Two distinct processes can be identified for the γ-Fe_2_O_3_ nanoparticles: a first process with a mass loss of 3.4% is visible in a temperature range between 344 and 444 °C, and a second one corresponding to a mass loss of 26.7% is taking place between 444 and 634 °C (see Figure 6A). According to the observed mass losses (see Table 2), these processes can be assigned to the transformation of γ-Fe_2_O_3_ to Fe_3_O_4_ and to the reduction of Fe_3_O_4_ to metallic Fe, respectively. It is worth noting the low temperature at which Fe_3_O_4_ reduces to metallic Fe which usually occurs at higher temperatures (T > 900 °C) [45,46]. This is because the nanoparticles have a large specific surface exposed to H_2_ gas which favors the reduction.

The stability of γ-Fe_2_O_3_ impregnated in H-USY (40) zeolite is shown in Figure 6B. The large porosity of Y zeolite makes sure that this material is full of gasses inside the porous; therefore, prior to the reduction, the nanoparticle impregnated zeolite has been degassed in a 0.5 atm He gas, as shown in Figure S5. During the heating process, the system losses 2.4% of the total mass and then, under cooling, it recovers 0.3% because part of the gasses go through the porous again. After degassing, the γ-Fe_2_O_3_ impregnated H-USY (40) zeolite is subjected to a reducing atmosphere and the TG curves show now several reduction processes that cannot be assigned undistinguishably to reduction from γ-Fe_2_O_3_ to Fe_3_O_4_ or from Fe_3_O_4_ to Fe due to the complexity of the system. Since γ-Fe_2_O_3_ nanoparticles are located both at the external surface and inside the mesopores of the zeolite, the exposition to the reducing gas is different: the nanoparticles at the surface of the zeolite will be reduced at similar temperatures to those of the nanoparticles alone (see Figure 6A), whereas the nanoparticles inside the mesopores are protected by the zeolite and, consequently, require higher temperature and more time to be reduced. This argument is supported by the fact that even at 900 °C the reduction process continues still for several minutes. It is assumed that this last process is the reduction to metallic Fe.

Assuming that the whole reduction process from 50 to 900 °C corresponds to the mass reduction from γ-Fe_2_O_3_ to metallic Fe, the concentration of γ-Fe_2_O_3_ nanoparticles can be calculated. The mass loss in this temperature range is 2.26 mg and it corresponds to the 30% of oxygen mass loss in this reduction process (see Table 2); therefore, there are 7.53 mg of γ-Fe_2_O_3_. Considering the total mass 62.66 mg, the magnetic nanoparticle concentration in the zeolite is 12%. This value is in agreement with the estimated volume of the magnetic colloid dropped on the zeolite for the impregnation.

**Figure 7:**
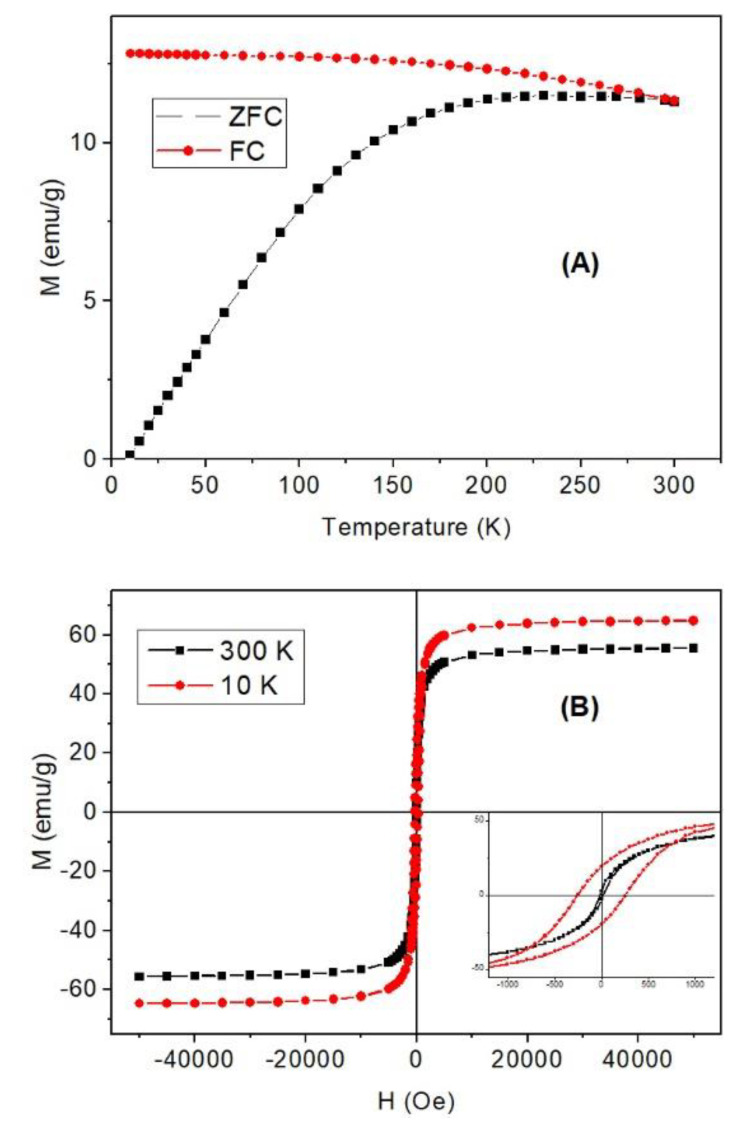
(**A**) ZFC-FC curves at 100 Oe and (**B**) hysteresis cycles at 10 and 300 K. The inset shows a detail of the curves at low field.

The ZFC-FC and hysteresis curves (Figure 7A) show that the nanoparticles of 11 nm have a blocking temperature around room temperature, i.e., the nanoparticles are at the limit of superparamagnetism-ferromagnetism, which is the most efficient condition for induction heating [27]. The saturation magnetization is 65 emu/g at 10 K and 55 emu/g at 300 K, and these values are the expected ones for 11 nm nanoparticles (Figure 7B). On the other hand, the coercivities are 300 and 15 Oe at low and high temperature, respectively. The small coercive field at room temperature is related to the size distribution and the high blocking temperature: most of the particles are superparamagnetic but there exists a contribution from the largest ones which have ferromagnetic behavior.

**Figure 8:**
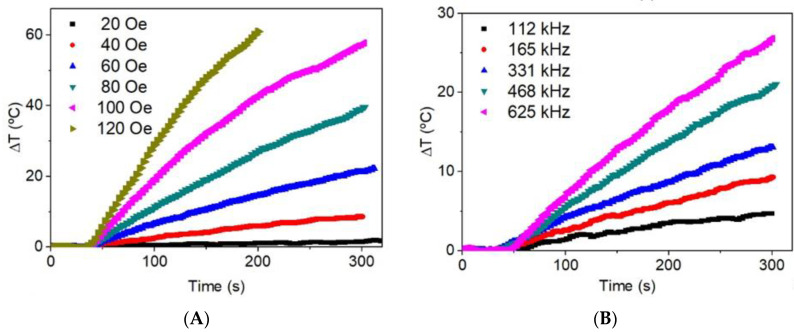
Heating curves (**A**) as a function of the applied magnetic field at f = 331 kHz (above) and (**B**) as a function of the frequency at H = 52 Oe (below).

**Figure 9:**
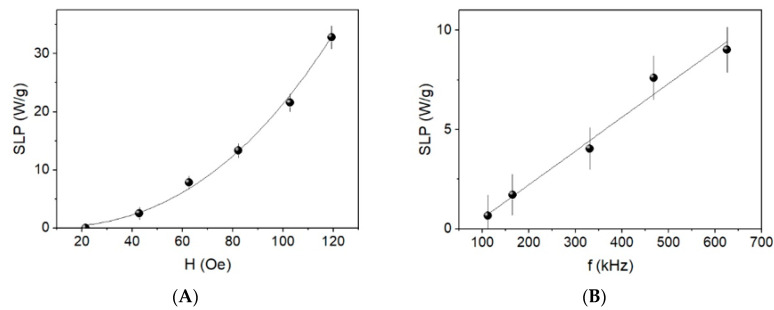
Specific loss power (SLP) values (**A**) as a function of the field at f = 331 kHz (above) and (**B**) as a function of the frequency at H = 52 Oe (below).

The heating efficiency of the colloidal nanoparticles is calculated by means of the slope of the heating curves (Equation (1)) as a function of the field amplitude and frequency (Figure 8). The SLP values, calculated from the slope of these curves, are shown in Figure 9. As can be seen, SLP depends on the square of field amplitude whereas it is lineal with the frequency. These behaviors suggest that the SLP of the magnetic colloid has the behavior predicted by the linear response theory (LRT), i.e., the condition μ_0_M_s_VH_0_ ≤ k_B_T is satisfied, which is the case for the 11 nm nanoparticles and the maximum field applied H_0_ [47]. Under these conditions SLP ∝ fH_0_^2^, as observed for the SLP curves in Figure 8.

The temperature increase of the γ-Fe_2_O_3_ nanoparticle impregnated zeolite is about 60 °C in 180 s, as shown in Figure 8. The hydrocracking process requires temperatures above 200 °C [48,49]; therefore, the iron oxide nanoparticles produces an increase temperature well below the required one. However, the γ-Fe_2_O_3_ nanoparticles have to be thought as a triggering process for the production of metallic Fe nanoparticles inside the zeolite when H_2_ gas fluxes through the reactor. The metallic Fe nanoparticles have a saturation magnetization about 200 emu/g at room temperature, i.e., four time higher than the γ-Fe_2_O_3_ nanoparticles and, consequently, a much higher temperature can be reached in a shorter time. The pre-impregnation of zeolite with γ-Fe_2_O_3_ instead of Fe nanoparticles have several advantages: (i) the smaller saturation magnetization of the γ-Fe_2_O_3_ reduces the aggregation degree and allows a much more homogeneous distribution of nanoparticles inside the zeolite, (ii) the Fe nanoparticles exposed to air normally oxidizes to non-magnetic hematite or wustite giving place to a core-shell structure that can decreases the heating efficiency of the metallic Fe [50]. Therefore, the γ-Fe_2_O_3_ nanoparticles have to be considered as a first step to activate, under H_2_ flux, the reduction to Fe nanoparticles, and this last one can reach the enough temperature to induce the catalytic process.

The measurements with the optical probe are performed by placing it in the middle of the colloidal volume, which assures the measurement of the mean temperature of the system. For the measurement of the nanoparticle impregnated zeolite it is necessary to use an infrared camera. The infrared camera measures the temperature of the surface where the heat exchange with the surrounding is maximum. To know which is the reliability of the measurements, the magnetic colloid has been measured with the optical probe and the infrared camera in order to compare these values.

**Figure 10:**
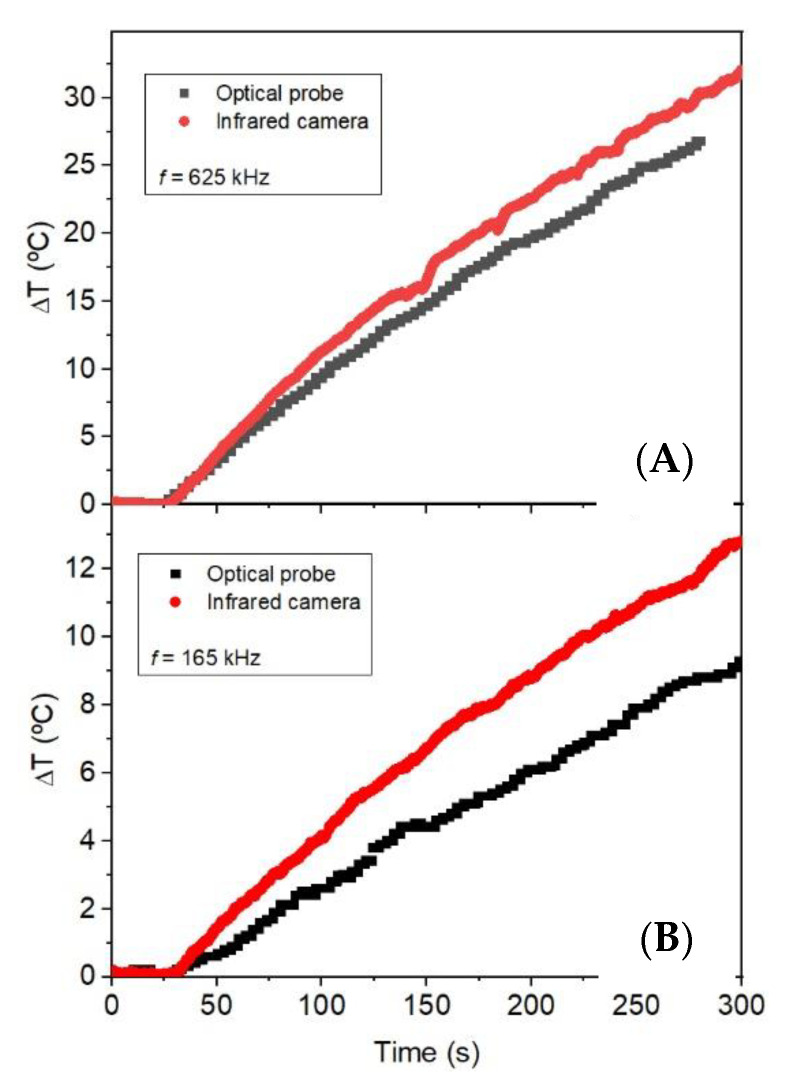
Heating curves at H = 52 Oe and at (**A**) f = 625 kHz (above) and (**B**) f = 165 kHz (below). The data were recorded by optical probe (black line) and infrared camera (red line).

Figure 10 shows the heating curves at 165 and 468 kHz with H = 52 Oe measured simultaneously with optical fiber and infrared camera. It is observed that the heating rate is different for both kinds of measurements; the heating rate is higher when measured by the camera than by the optical probe. The difference in SLP values at low frequency is higher than at high frequency. This is due to the measuring setup: thermal probe measures the average temperature of the fluid, while the thermal camera measures the temperature at the liquid–air interface. The heat capacity of the air (C_a_ = 1 J/gK) is four times smaller than that of water (C_w_ = 4.185 J/gK), and this means that, for the same thermal energy transfer, the temperature increase in water is smaller than in air. When the heating rate of the liquid is low, the air heats up “apparently” faster than water, but it is only due to the lower heat capacity of air. When the heating efficiency is higher, as in the case of 625 kHz, the temperature increase is similar for both sensors.

**Figure 11:**
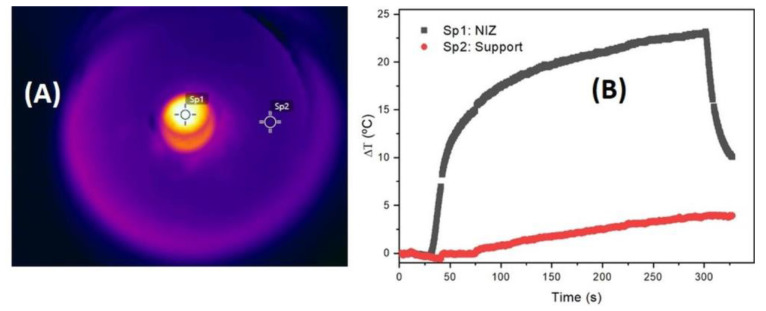
(**A**) Image of the infrared camera measured at two different measuring points: in the sample (Sp1) and in a middle point between the coils and the sample (Sp2) after 300 s of applied field is on. (**B**) Temperature increase versus time for nanoparticle impregnated H-USY (40) zeolite (NIZ, black line) and for the sample chamber Sp2 (red line).

The heating rate of the nanoparticles impregnated H-USY (40) zeolite subjected to a field of 331 kHz and 120 Oe is shown in Figure 11. The temperature increase of the sample holder is also added to check that the temperature increase comes from the sample and not from the heat produced by the coils. In Figure 11A, a real-time measurement of the infrared camera with different measurement points can be seen, the spot Sp1 is the heat released by the nanoparticles impregnated zeolite whereas the spot Sp2 is in the middle between the coils and the sample. As can be seen in Figure 11B, at the first seconds when the slope is measured, the temperature increase in S2 is negligible; therefore, the temperature increase is related to the heat transport from the nanoparticles to the zeolite. Unfortunately, it is not possible to determine the SLP of the nanoparticle impregnated zeolite because the specific heat of the material is unknown. However, it is possible to compare the heating efficiency of the nanoparticles with the nanoparticle impregnated zeolite by comparing the temperature increase rate per iron mass concentration (ΔT/Δt)/c_Fe_. The Fe concentration in the aqueous colloid is c_w_ = 6.0 ± 0.1 wt.% whereas the Fe concentration in the zeolite is c_z_ = 8.4 ± 0.1 wt.%, thus giving the values 7.8 ± 0.1 and 7.6 ± 0.1 K/s for the aqueous colloid and the nanoparticles impregnated zeolite, respectively. As can be seen, both values are similar inside the experimental errors. This suggests that the 11 nm γ-Fe_2_O_3_ nanoparticles inside a solid matrix such as zeolite are able to release heat as efficiently as in a liquid medium. This is because the Brownian relaxation at this size is negligible.

All these results have shown that γ-Fe_2_O_3_ magnetic nanoparticles, when subjected to radiofrequency fields, are able to release heat even when they are immobilized in a matrix making the γ-Fe_2_O_3_ impregnated H-USY zeolite a good candidate for catalytic processes under heating induction.

## 4. Conclusions

The nanoparticle impregnated H-USY (15) and H-USY (40) zeolites have been successfully synthesized. The XRD and TEM results show that the γ-Fe_2_O_3_ nanoparticles of 11 nm in size are mostly on the surface of H-USY (15), whereas in the case of H-USY (40) there are particles on the surface and inside of the 26 nm mesoporous. These results are supported by the studies of textural properties of parent H-USY zeolites and the corresponding nanoparticle impregnated zeolites evaluated by N_2_ adsorption. These measurements show a decrease of the external area and of the porous volume for both Y zeolites, but the observed effect is larger for the H-USY (15), indicating that there are more particles on the surface of H-USY (15) than on H-USY (40). Besides, the acidity is higher for the H-USY (15) because of the larger ratio of Al/Si sites, but the addition of magnetic nanoparticles does not affect considerably the acidity of the catalysts, neither in terms of number nor of the strength of the centers.

The TG characterization of γ-Fe_2_O_3_ nanoparticles under H_2_ atmosphere reveals that γ-Fe_2_O_3_ can be completely reduced to metallic Fe at T = 634 °C, a temperature much smaller than the one corresponding to the bulk reduction; this is due to the high specific surface of the nanoparticles that can be exposed to H_2_ and facilitates the reduction process. On the other hand, the TG analysis of the nanoparticle impregnated zeolite exhibits a more complex behavior with a higher reducing temperature (T ~ 900 °C). This is in agreement with nanoparticles on the surface, that can be easily reduced, and inside the zeolite that hinder the access to the H_2_.

The magnetic properties of the γ-Fe_2_O_3_ show that the nanoparticles have a blocking temperature around 300 K, which means that these nanoparticles are at the limit of superparamagnetic-ferromagnetic behavior, a condition for a most efficient induction heating. The heating induction experiment performed at of 331 kHz and 120 Oe shows the heating release of the colloid and the impregnated zeolite characterized by (ΔT/Δt)/c_Fe_ are quite similar, making this material combining magnetic and acidic catalytic properties very appropriate for catalytic reactions under radiofrequency fields.

Despite achieving temperatures below those requires for hydrocracking process, the obtained results represent an important kick off for an innovative energy saver hydrocracking process thanks to a local heating system provided by in situ generated iron-nanoparticles and a radiofrequency electromagnetic field. The application in the catalytic cracking of waste plastics under hydrogen atmosphere seems highly promising since it can trigger the conversion of the γ-Fe_2_O_3_ nanoparticles to metallic Fe. This is a very important aspect since the metallic Fe nanoparticles have a saturation magnetization four times higher than the γ-Fe_2_O_3_ nanoparticles and, consequently, a much higher temperature can be reached. In, this way the high energetic demands of the cracking process are expected to be strongly reduced thanks to an innovative local heating system provided by in situ generated iron-nanoparticles.

## Figures and Tables

**Table 1:** Textural and acidic properties of parent and nanoparticles-impregnated H-USY (15) and H-USY (40) zeolites.

Catalyst	S_ext_ [m^2^/g]	V_micro_ [cm^3^/g]	V_meso_ [cm^3^/g]	V_total_ [cm^3^/g]	Weak Acid Sites [umol/g]	Strong Acid Sites [umol/g]	Total Acidity [umol/g]
H-USY (15)	192	0.25	0.23	0.48	332	319	651
NPs/H-USY (15)	160	0.19	0.21	0.40	330	294	627
H-USY (40)	251	0.21	0.26	0.46	55	182	237
NPs/H-USY (40)	238	0.17	0.26	0.43	46	162	208

**Table 2:** Reduction process, calculated and experimental mass loss and temperature range of the TG measurement in γ-Fe_2_O_3_ nanoparticles (NPs) and nanoparticles impregnated H-USY (40) zeolite (NIZ).

System	Reduction Process	Mass Loss (Calculated)	Mass Loss (Experimental)	Temperature Range
NPs	3Fe_2_O_3_ + H_2_ → 2Fe_3_O_4_ + H_2_O	3.3%	3.4%	344–444 °C
	Fe_3_O_4_ + 4H_2_ → 3Fe + 4H_2_O	27.6%	27.6%	444–634 °C
NIZ	Fe_2_O_3_ + 3H_2_ → 2Fe + 3H_2_O	30.0%	30.3%	50–900 °C

## Supplementary Materials

The following are available online at https://www.mdpi.com/1996-1944/13/18/4013/s1, Figure S1: XRD diffraction pattern of γ-Fe2O3 nanoparticles, Figure S2: TEM image of the H-USY (40), Figure S3: TEM images of nanoparticles impregnated zeolite H-USY (40), Figure S4: TEM images of nanoparticles impregnated zeolite H-USY (40) at different augments, Figure S5 (A): Degassing of the nanoparticles impregnated zeolite H-USY (40), (B) Change of mass rate on heating (red arrow) and cooling (blue arrow).
