# Supplementary material for: Induction Heating in Nanoparticle Impregnated Zeolite
Source: Materials (Basel). 2020 Sep 10;13(18):4013. doi: 10.3390/ma13184013 (PMC7558316; doi:10.3390/ma13184013)
Supplement: Supplementary file 1 [file materials-13-04013-s001.pdf]

materials

MDPI

Supplementary Materials

Induction Heating in Nanoparticle Impregnated Zeolite

Irene Morales, Marta Muñoz, Catia S. Costa, Jose Maria Alonso, João Miguel Silva, Marta Multigner, Mario Quijorna, M. Rosário Ribeiro and Patricia de la Presa

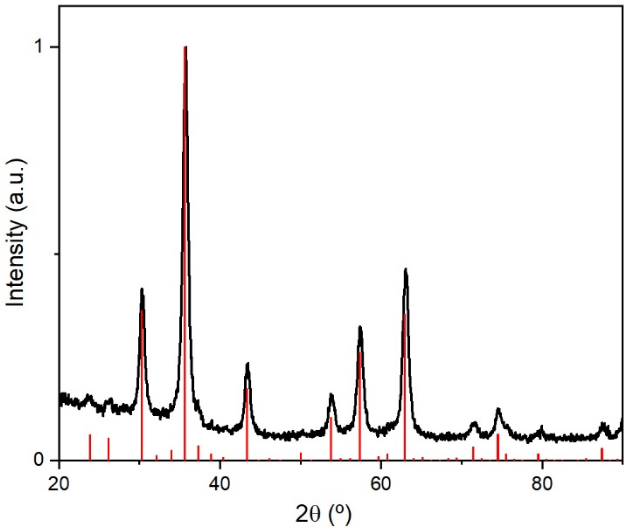

Figure S1. Experimental XRD diffraction pattern of γ-Fe_2O_3 nanoparticles (black lines). The red bars are the peaks and intensity of the calculated diffraction pattern according to ICSD 01-089-5892.

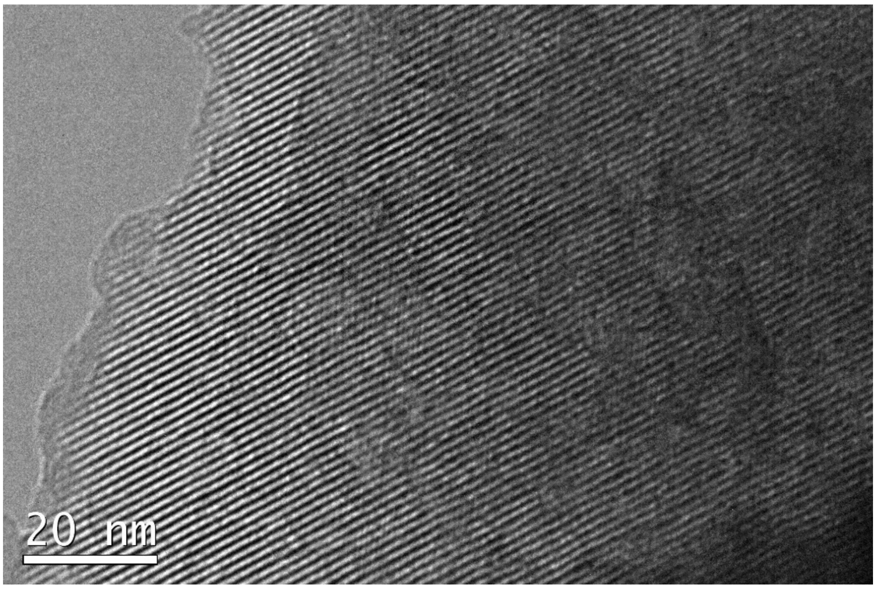

Figure S2. TEM image of the H-USY (40). Lighter zones corresponding to mesopores and the sodalite structure are observed.

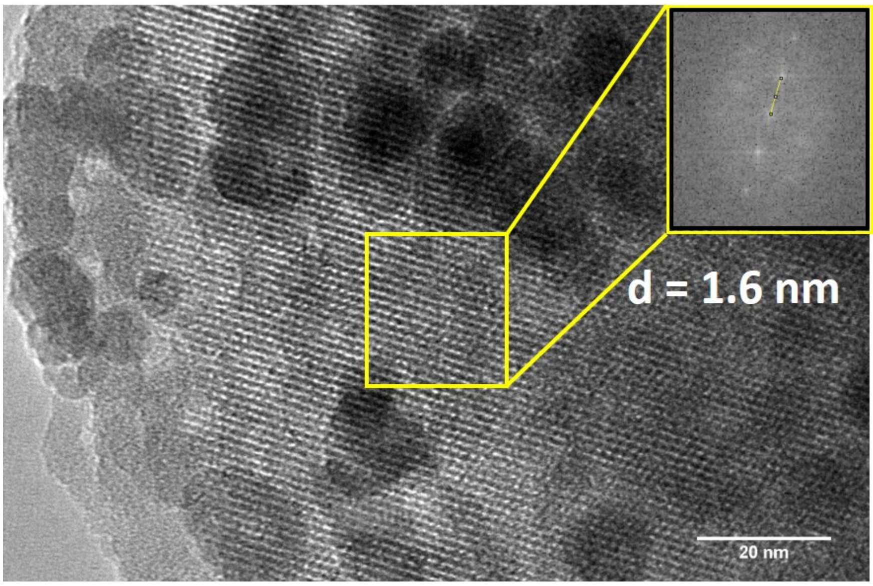

Figure S3. TEM image of the nanoparticles impregnated zeolite H-USY (40). The lighter lines are separated by 1.6 nm and correspond to the spherical supercages showing a diameter of 1.6 nm.

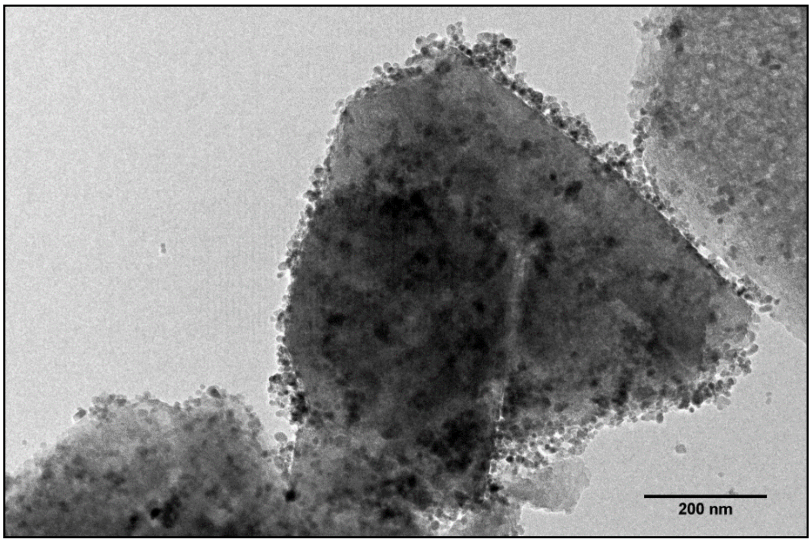

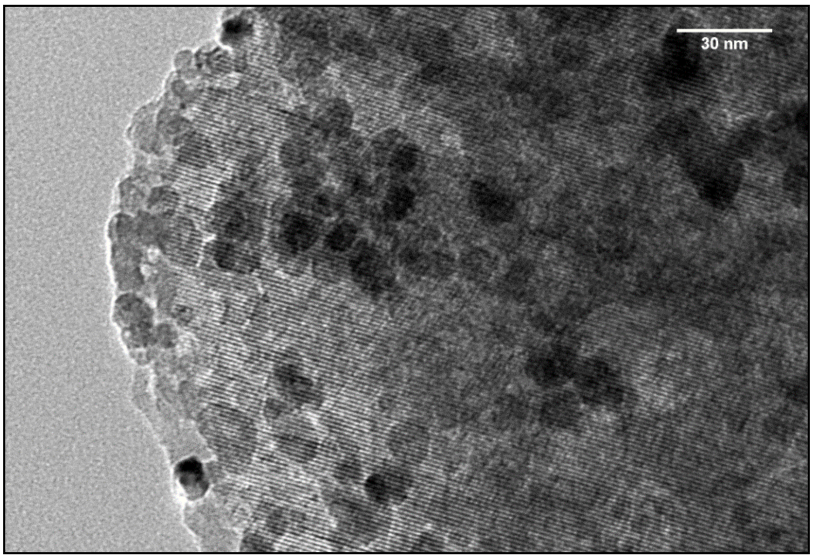

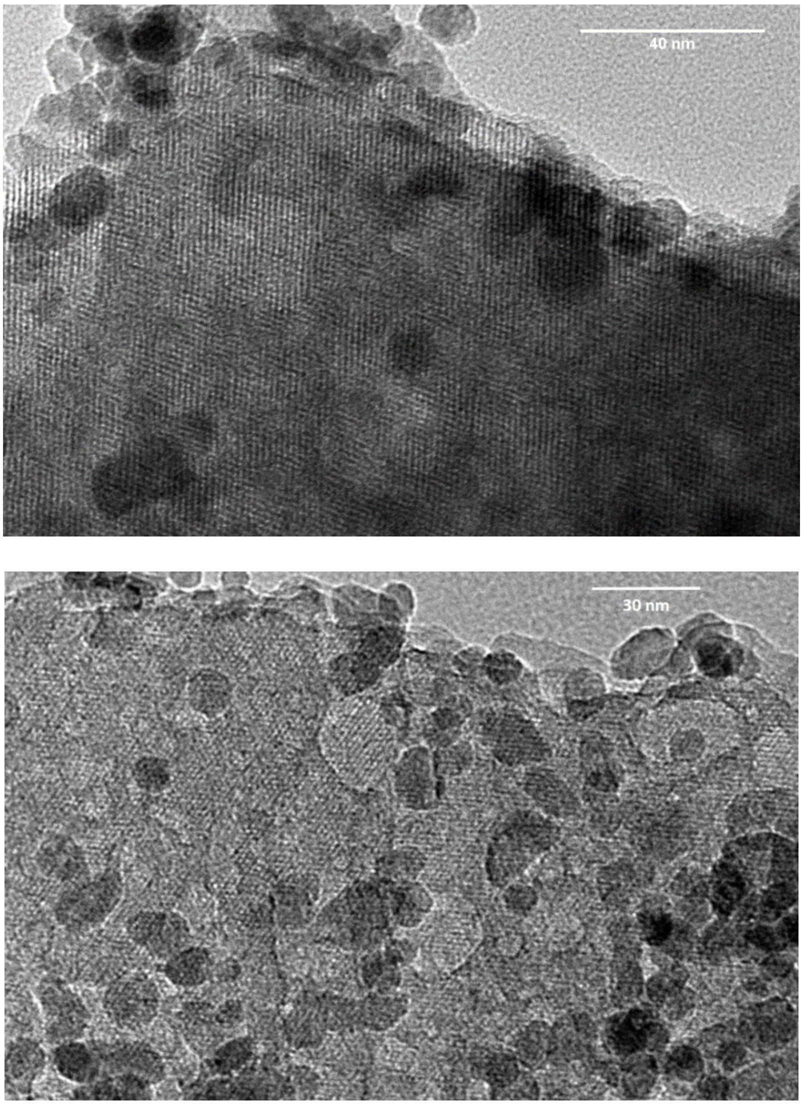

Figure S4. Nanoparticles impregnated zeolite H-USY (40) at different augments.

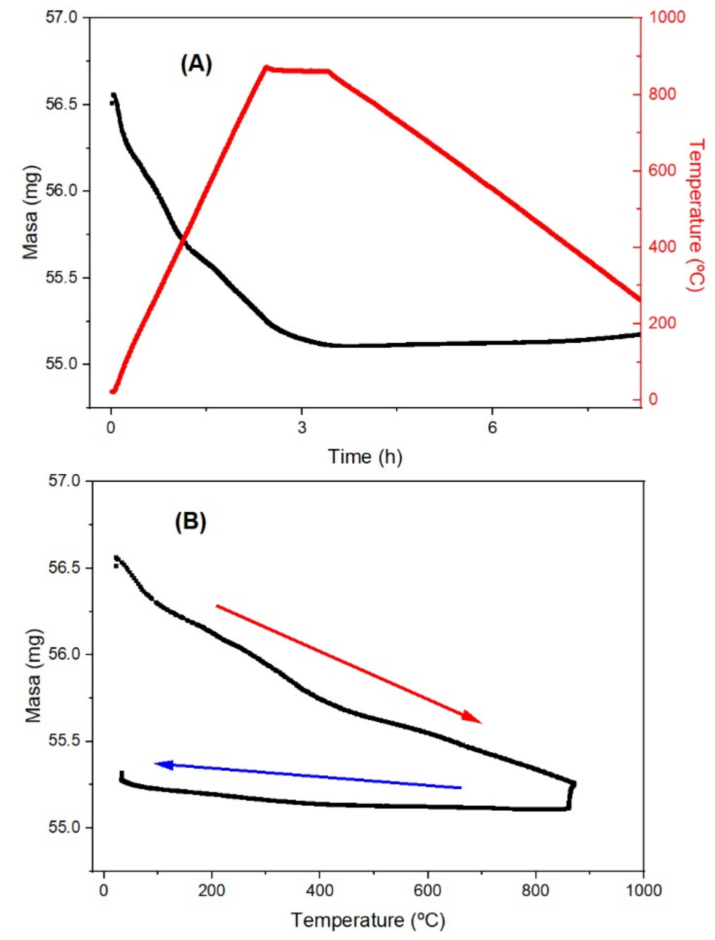

Figure S5. (**A**) Degassing of the nanoparticles impregnated zeolite H-USY40. (**B**) Change of mass rate on heating (red arrow) and cooling (blue arrow).

© 2020 by the authors. Submitted for possible open access publication under the terms and conditions of the Creative Commons Attribution (CC BY) license (http://creativecommons.org/licenses/by/4.0/).
